# Frequency of CAP-Defined Hepatic Steatosis and Its Association with Liver Fibrosis in Patients with Hepatitis Delta

**DOI:** 10.3390/v18101105

**Published:** 2026-10-07

**Authors:** Çiğdem Mermutluoğlu, Saim Dayan, Yakup Demir, Ayşe Özlem Mete, Mustafa Kemal Çelen

**Affiliations:** 1 Department of Infectious Diseases and Clinical Microbiology, Dicle University, 21280 Diyarbakır, Turkeysaimdayan@hotmail.com (S.D.);; 2 Department of Infectious Diseases, Diyarbakır Memorial Hospital, 21070 Diyarbakır, Turkey; yakupdemir36@gmail.com; 3 Department of Infectious Diseases and Clinical Microbiology, Gaziantep University, 27310 Gaziantep, Turkey

**Keywords:** hepatitis delta virus, FibroScan, hepatic steatosis, controlled attenuation parameter, liver cirrhosis, liver stiffness measurement

## Abstract

Background and Aim: Hepatitis delta virus (HDV) infection is among the most aggressive forms of chronic viral hepatitis: fibrosis advances quickly, and cirrhosis, hepatocellular carcinoma (HCC) and hepatic decompensation develop more often than with other hepatitis viruses. Metabolic dysfunction-associated steatotic liver disease (MASLD) is now the leading chronic liver disorder worldwide, and it may add to liver damage when it occurs alongside viral hepatitis. To date, however, data on hepatic steatosis in patients with chronic HDV infection remain scarce. We therefore aimed to describe the frequency of hepatic steatosis, assessed by the controlled attenuation parameter of transient elastography (FibroScan), in patients with chronic HBV infection and persistent anti-HDV seropositivity, and to examine whether it was associated with liver fibrosis. Methods: A total of 125 patients with chronic HBV infection and anti-HDV antibodies documented for at least six months were enrolled in this cross-sectional study between March and September 2024. HDV-RNA was tested in all participants. FibroScan provided liver stiffness measurement (LSM, kPa) and the controlled attenuation parameter (CAP, dB/m). We used CAP ≥ 248 dB/m to identify steatosis and LSM ≥ 12.5 kPa to identify cirrhosis. Because cardiometabolic risk factors were not systematically recorded, the criteria for MASLD could not be applied, and steatosis is reported as CAP-defined hepatic steatosis. Multivariable logistic regression was used to look for factors linked to LSM-defined advanced fibrosis (LSM ≥ 9.5 kPa), with sensitivity analyses accounting for ALT elevation. Results: The mean age was 39.0 years, and 74.4% (*n* = 93) of patients were male. HDV-RNA was detectable in 68.0% (*n* = 85). Of the 85 patients previously treated with pegylated interferon-alpha, 26 (30.6%) had a sustained virological response (SVR). Median LSM was 10.1 kPa (IQR 9.1–10.7) and median CAP 218 dB/m (IQR 211–239); cirrhosis was present in 15.2% (*n* = 19). CAP-defined steatosis was found in 24.0% (*n* = 30). Patients with and without steatosis were similar in age, sex, HDV-RNA status, liver stiffness, cirrhosis rate and SVR rate (36.4% vs. 28.6%, *p* = 0.593). After adjustment, detectable HDV-RNA was associated with advanced fibrosis (adjusted odds ratio [aOR] 4.89, 95% CI 2.13–11.26, *p* < 0.001), including after further adjustment for ALT. No significant association was found for steatosis (aOR 0.82, 95% CI 0.32–2.12, *p* = 0.684), although the confidence interval was wide. Conclusions: About one in four anti-HDV-positive patients had CAP-defined hepatic steatosis. Detectable HDV-RNA was associated with LSM-defined advanced fibrosis, whereas no significant association with steatosis was found; the precision of this estimate was limited, and the exploratory cirrhosis analysis was underpowered. Whether steatosis in this setting reflects metabolic dysfunction, and whether it affects long-term outcomes, warrants prospective study with full metabolic phenotyping.

## 1. Introduction

Hepatitis delta virus (HDV) is a small, defective RNA virus. It cannot assemble without the hepatitis B surface antigen (HBsAg), so infection is confined to people who carry hepatitis B virus (HBV), acquired either together with HBV (co-infection) or on top of an established HBV infection (superinfection) [1]. Estimates of the global burden vary widely. One meta-analysis suggested that 62–72 million people may be anti-HDV positive [2], whereas another found anti-HDV antibodies in about 4.5% of HBsAg-positive individuals, corresponding to roughly 12 million people [3]. More recent modelling by the Polaris Observatory indicates that earlier figures probably overestimated the number of people with active infection [4]. The same analysis, covering 25 countries and territories, showed marked geographic variation, with the highest prevalence in Mongolia [4].

Compared with HBV monoinfection, HDV roughly triples the rate of progression to cirrhosis and doubles the risk of HCC [5]. Detectable HDV-RNA has been linked independently to advanced fibrosis and cirrhosis in cohort studies, and high HDV-RNA levels predict progression to cirrhosis and liver cancer [6]. Turkey is recognized as an endemic region for both HBV and HDV infection; a meta-analysis published in 2024 reported an anti-HDV seroprevalence of 3.37% among HBsAg-positive blood donors and considerably higher rates among patients with established cirrhosis [7]. The southeastern Anatolian region has historically demonstrated among the highest rates of HBV/HDV co-infection in Turkey [7,8], positioning Diyarbakir as a clinically relevant study site.

The metabolic side of this picture is less clear. Chronic HBV infection does not seem to make fatty liver more likely; several studies have instead reported an inverse association between HBV infection, or HBV viraemia, and fatty liver, possibly reflecting interference of HBV with host lipid metabolism [9,10]. Because HDV can only replicate in HBsAg-positive hosts, we do not know whether this pattern holds once HDV is added, or whether steatosis adds meaningfully to liver injury in these patients.

Metabolic dysfunction-associated steatotic liver disease (MASLD) is present in roughly one in three adults worldwide [11,12], making it the main cause of chronic liver disease today. Its inflammatory form, metabolic dysfunction-associated steatohepatitis (MASH), can progress to fibrosis, cirrhosis and HCC in its own right. The term MASLD was introduced in 2023 by a multisociety Delphi consensus to replace the older non-alcoholic fatty liver disease (NAFLD) terminology [13].

With vibration-controlled transient elastography (VCTE; FibroScan, Echosens, Paris, France), liver fibrosis and steatosis can be assessed at the same visit, through liver stiffness measurement (LSM, kPa) and the controlled attenuation parameter (CAP, dB/m), respectively; current international guidelines recommend it as a first-line non-invasive test [14,15]. Most FibroScan studies in HDV infection have, however, focused on liver stiffness alone [16], and data on CAP-defined steatosis in this population are sparse. The question has become more relevant now that bulevirtide can suppress HDV replication [17]: if metabolic liver injury persists after the viral component is controlled, it could contribute to residual fibrosis or cancer risk. We therefore used LSM and CAP to estimate the frequency of steatosis in a cohort of patients with chronic HBV infection and persistent anti-HDV seropositivity from a high-prevalence region, and to test whether steatosis was associated with LSM-defined fibrosis after accounting for age, sex and HDV-RNA status.

## 2. Materials and Methods

### 2.1. Study Design and Patient Selection

We carried out this cross-sectional study in the Department of Infectious Diseases, Dicle University Faculty of Medicine Hospital, in Diyarbakir, Turkey. The local ethics committee approved the protocol (approval no. TIP.23.022), the study followed the Declaration of Helsinki, and every participant gave written informed consent. Between March and September 2024, 125 patients with anti-HDV antibodies were enrolled.

Adults (≥18 years) with anti-HDV positivity documented for at least six months were eligible, provided they gave consent to take part. In this study, “chronic HDV infection” refers to chronic HBV infection with anti-HDV positivity documented for at least six months; it does not imply current viraemia, which was present in 85 of the 125 patients.

We excluded patients with hepatitis C or HIV co-infection, a previous liver transplant, or alcohol intake above 140 g/week (women) or 210 g/week (men), as well as those in whom a reliable examination was not possible, for example, because of ascites on the day of FibroScan. Patients with a history of hepatocellular carcinoma or hepatic decompensation were not excluded if a valid examination could be obtained, and no patient was excluded on the basis of ALT levels.

### 2.2. Clinical and Laboratory Assessment

HDV-RNA was measured in every patient during routine care in the Molecular Diagnostics Laboratory of the Department of Medical Microbiology, Dicle University Faculty of Medicine, using the same real-time RT-PCR assay throughout the study (RoboGene HDV RNA Quantification Kit, AJ Roboscreen, Leipzig, Germany). Results were available to us only as detectable or undetectable relative to the lower limit of detection of this assay (10 IU/mL); viral load values were not available. We regarded ALT as raised above 25 U/L for women and 35 U/L for men, the upper limits of normal recommended in the AASLD hepatitis B guidance [18]. We noted whether patients were taking an oral nucleos(t)ide analogue (NA) for HBV and whether they had ever received pegylated interferon-alpha (peg-IFN-α). Peg-IFN-α had been given for at least 12 months and had been completed before the FibroScan examination; no patient was receiving interferon at the time of assessment. The interval between the end of treatment and FibroScan varied between patients and was not recorded. SVR was defined in this study as undetectable HDV-RNA at a post-treatment assessment performed at least six months after the end of peg-IFN-α therapy, using the same assay; a negative HDV-RNA result in someone never given interferon was not counted as SVR. A history of hepatic decompensation (ascites, variceal bleeding or hepatic encephalopathy) and of hepatocellular carcinoma, diagnosed according to EASL criteria, was obtained from clinical records. The study database did not systematically capture body mass index, waist circumference or other cardiometabolic risk factors.

### 2.3. FibroScan Protocol

All examinations were performed by a single certified operator (M.K.Ç.) after a fast of at least three hours, as currently recommended [19]. Probe choice depended on skin-to-capsule distance (M probe below 2.5 cm, XL probe at 2.5 cm or more). An examination was accepted as reliable if it included at least ten valid measurements, a success rate of 60% or higher and an IQR/median ratio no greater than 0.30 [14]. Because all measurements were made by the same operator, inter-operator reproducibility was not assessed. Results were expressed in kPa for LSM and dB/m for CAP. Patients were grouped into LSM-based categories using cut-offs commonly applied in chronic liver disease: below 7.0 kPa (presumed F0–F1), 7.0–9.4 kPa (presumed F2), 9.5–12.4 kPa (presumed F3) and 12.5 kPa or more (presumed F4, cirrhosis) [14,20]. These categories are indirect estimates and are not equivalent to histological stages; the cut-offs were derived largely in other chronic liver diseases and have not been validated in HDV infection. Accordingly, “advanced fibrosis” (LSM ≥ 9.5 kPa) and “cirrhosis” (LSM ≥ 12.5 kPa) in this study are LSM-defined outcomes. Steatosis was diagnosed at CAP ≥248 dB/m. This threshold for any steatosis (S ≥ 1) comes from an individual patient data meta-analysis in which most patients had chronic hepatitis B or C [21]. CAP may be less accurate in cirrhosis, and this threshold has not been validated specifically in HDV infection. Because cardiometabolic risk factors were not systematically available, the 2023 Delphi criteria for MASLD could not be applied. Patients with CAP ≥ 248 dB/m are therefore described as having CAP-defined hepatic steatosis. This term does not imply a metabolic cause: although excessive alcohol intake and other chronic liver diseases were excluded, steatosis related to HDV infection itself, to cirrhosis or to unmeasured factors cannot be ruled out. We did not attempt to diagnose steatohepatitis, which requires histology.

### 2.4. Statistical Analysis

Statistical analysis was done in R (version 4.6.0; R Foundation for Statistical Computing, Vienna, Austria). Figures were drawn with the matplotlib library (version 3.10.8; Python 3.12.3). Continuous data are summarised as mean ± SD or median (IQR), and categorical data as counts with percentages. Because continuous variables were not normally distributed (Shapiro–Wilk test), groups were compared with the Mann–Whitney U test; categorical variables were compared with Fisher’s exact test. Factors associated with LSM-defined advanced fibrosis (88 events) and cirrhosis (19 events) were examined by multivariable logistic regression. Covariates were chosen a priori: steatosis as the exposure of interest, age and sex as basic demographic confounders, and detectable HDV-RNA as the main virological determinant of liver injury; the small number of cirrhosis events did not allow a larger model. To address possible confounding by hepatic inflammation and treatment, the advanced fibrosis model was repeated with ALT elevation added, then with ALT elevation, previous peg-IFN-α therapy and current NA therapy added, and finally in patients with normal ALT only. SVR was not entered as a covariate because it is, by definition, part of HDV-RNA status. Because there were few cirrhosis events, the cirrhosis model was regarded as exploratory and was refitted with Firth’s penalised likelihood. In a sensitivity analysis, the models were repeated in patients with detectable HDV-RNA only. LSM was not compared between cirrhotic and non-cirrhotic patients, since cirrhosis was defined by LSM. Likewise, CAP was not compared statistically between patients with and without steatosis, since steatosis was defined by CAP. No formal sample size calculation was performed, and subgroup comparisons were not adjusted for multiple testing; they should be regarded as exploratory. We used a two-sided significance level of 0.05.

## 3. Results

### 3.1. Demographic Characteristics

The cohort comprised 125 patients (93 men, 32 women); their mean age was 39.0 years (SD 11.6), and HBsAg had been known to be positive for a median of 9 years (IQR 8–12). Anti-HDV positivity was confirmed in all participants, and HDV-RNA was requested for each. HDV-RNA was detectable in 85 patients (68.0%) and undetectable in 40 (32.0%). Twenty patients (16.0%) were receiving oral NA therapy, and ALT was above the upper limit of normal in 55 (44.0%). Characteristics according to steatosis status are shown in Table 1.

### 3.2. Treatment Status and SVR

Sixty-eight percent of patients (*n* = 85) had received peg-IFN-α for at least 12 months; the remaining 32% (*n* = 40) had never received interferon. Of the 85 treated patients, 26 (30.6%) had achieved SVR. SVR rates were similar in patients with and without steatosis (8 of 22 [36.4%] vs. 18 of 63 [28.6%], *p* = 0.593). Viewed from the other direction, 8 of the 26 patients with SVR (30.8%) and 14 of the 59 without SVR (23.7%) had steatosis, as shown in Table 2; both sets of figures derive from the same 2 × 2 table. Compared with non-responders, patients with SVR had lower LSM (median 8.6 vs. 10.4 kPa, *p* < 0.001), less often had LSM ≥ 9.5 kPa (42.3% vs. 81.4%, *p* = 0.001) and rarely had elevated ALT (3.8% vs. 59.3%, *p* < 0.001) (Table 2). Fourteen of the 40 patients who had never received interferon had undetectable HDV-RNA; they were not counted as having SVR.

### 3.3. FibroScan Findings

**Figure 1:**
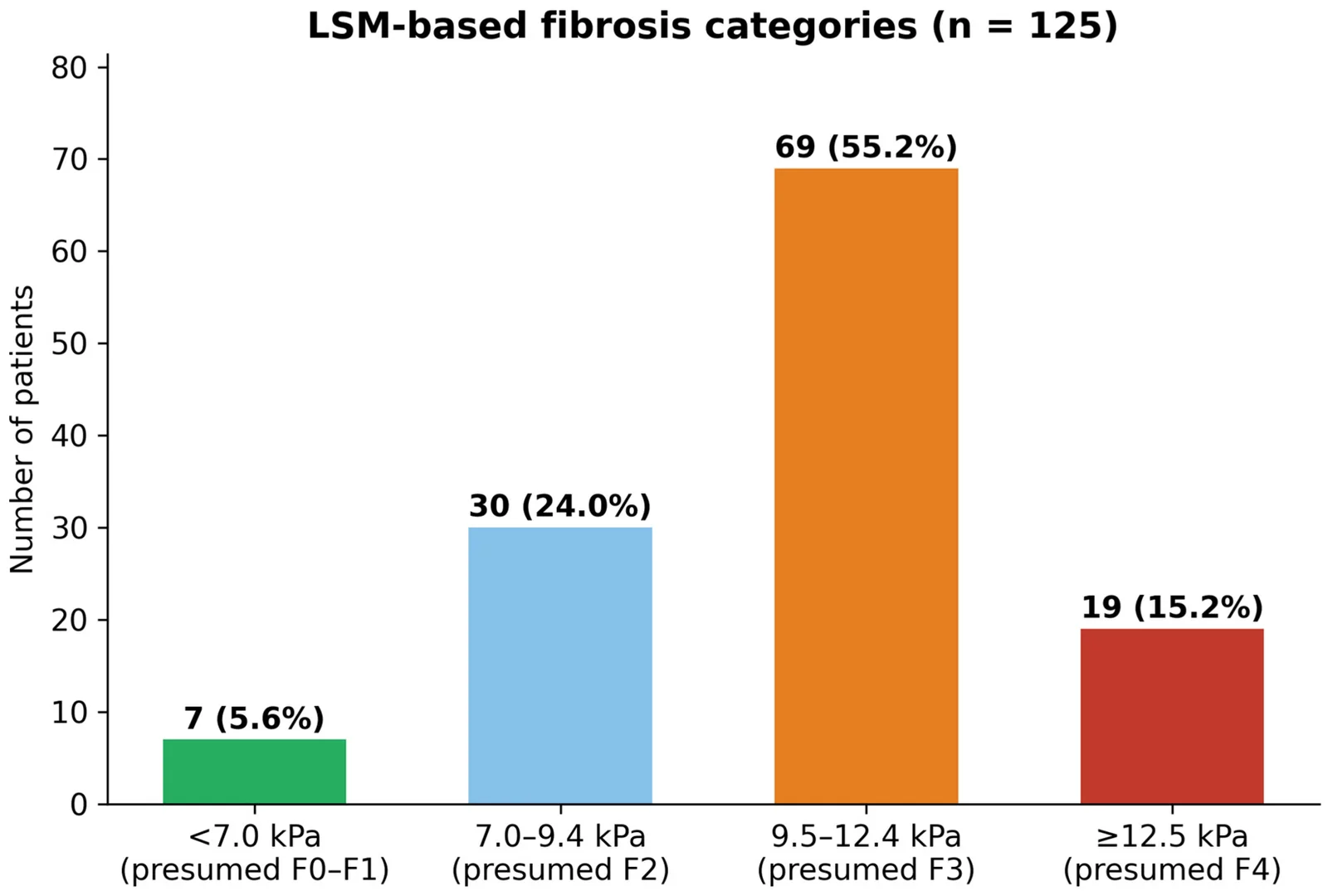
Liver stiffness measurement (LSM)-based fibrosis categories in 125 patients with chronic HDV infection. Presumed fibrosis stages are shown in parentheses; they are indirect estimates, not histological stages.

**Figure 2:**
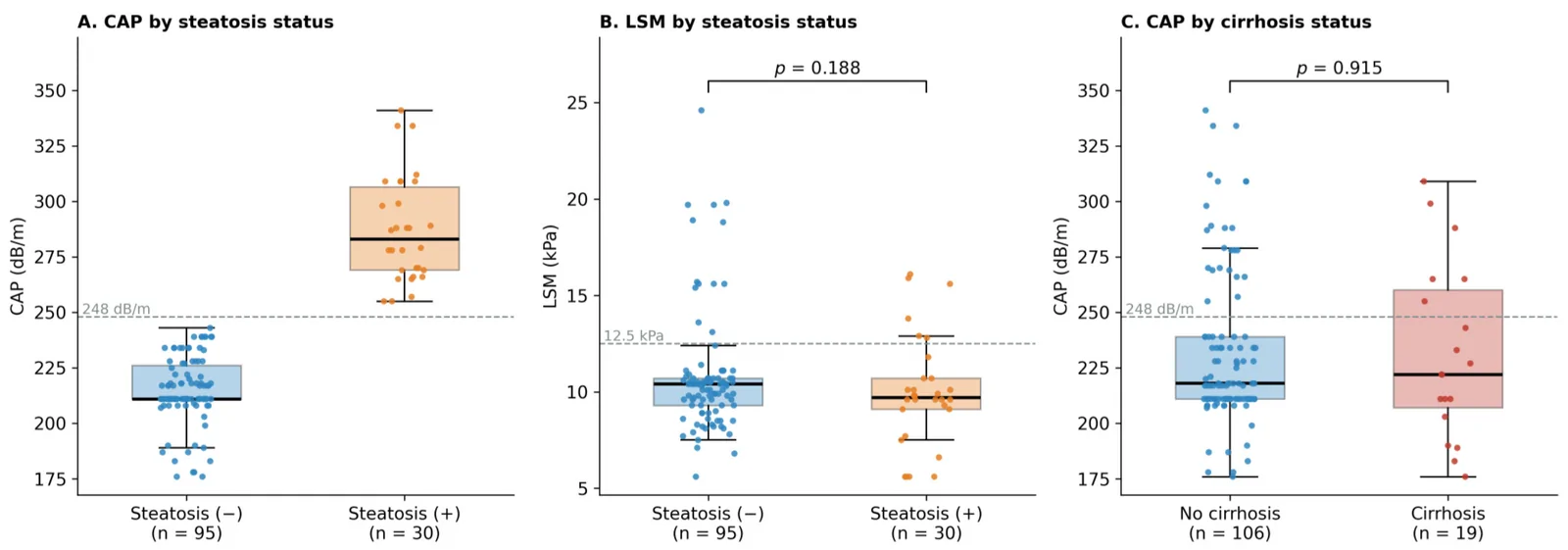
(**A**) Controlled attenuation parameter (CAP) and (**B**) LSM according to steatosis status; (**C**) CAP according to cirrhosis status. Each box marks the median and IQR, with whiskers reaching 1.5 × IQR; each dot represents one patient (horizontal jitter for visibility only). Dashed lines mark the 248 dB/m steatosis and 12.5 kPa cirrhosis thresholds. *p* values (panels (**B**,**C**)): Mann–Whitney U test; CAP was not compared statistically in panel (**A**) because it defines the groups.

Across all patients, mean LSM was 10.6 kPa (SD 3.1; median 10.1, IQR 9.1–10.7) and mean CAP was 232.1 dB/m (SD 35.7). In the LSM-based categories, 7 patients (5.6%) had values below 7.0 kPa (presumed F0–F1), 30 (24.0%) 7.0–9.4 kPa (presumed F2), 69 (55.2%) 9.5–12.4 kPa (presumed F3) and 19 (15.2%) 12.5 kPa or more (presumed F4) (Figure 1). Mean LSM was 16.5 kPa in patients with cirrhosis and 9.5 kPa in those without; because cirrhosis was defined by LSM, this difference was not tested. CAP was similar in patients with and without cirrhosis (231.7 vs. 232.2 dB/m, *p* = 0.915; Figure 2C).

### 3.4. Cirrhosis, Clinical Events and Factors Associated with Fibrosis

**Figure 3:**
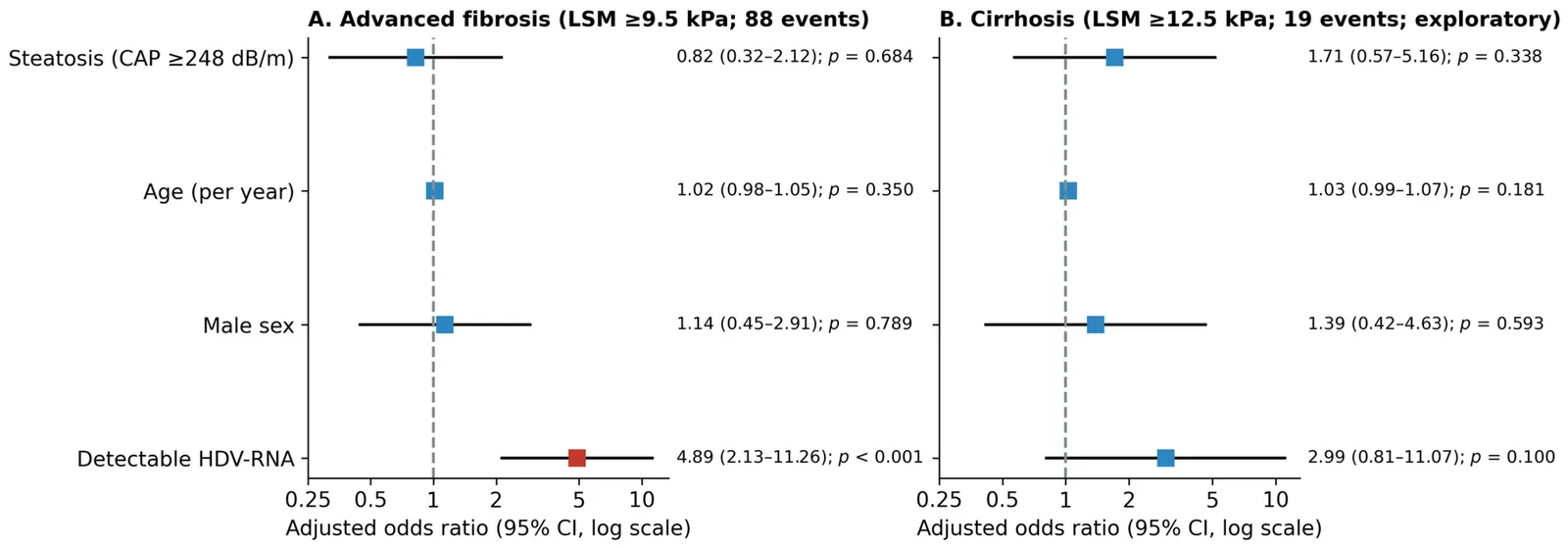
Multivariable logistic regression for (**A**) advanced fibrosis (LSM ≥ 9.5 kPa) and (**B**) cirrhosis (LSM ≥ 12.5 kPa). Squares mark adjusted odds ratios, with 95% CIs drawn as horizontal lines (log scale); red indicates *p* < 0.05.

LSM-defined cirrhosis was present in 19 patients (15.2%). According to clinical records, 8 patients (6.4%) had a history of hepatic decompensation and were being evaluated for liver transplantation, and 2 (1.6%) had been diagnosed with HCC. Patients with cirrhosis were more often receiving oral NA therapy (36.8% vs. 12.3%, *p* = 0.014). In multivariable analysis (Table 3 and Figure 3), detectable HDV-RNA was independently associated with advanced fibrosis (aOR 4.89, 95% CI 2.13–11.26, *p* < 0.001), and no significant association was found for steatosis, age or sex. In the exploratory cirrhosis model, no variable was significantly associated with cirrhosis (steatosis: aOR 1.71, 95% CI 0.57–5.16, *p* = 0.338; detectable HDV-RNA: aOR 2.99, 95% CI 0.81–11.07, *p* = 0.100), and the Firth-corrected model gave similar estimates. The association between detectable HDV-RNA and advanced fibrosis persisted after adjustment for ALT elevation (aOR 4.12, 95% CI 1.45–11.72), after additional adjustment for previous peg-IFN-α and current NA therapy (aOR 4.22, 95% CI 1.47–12.07), and among the 70 patients with normal ALT (aOR 4.83, 95% CI 1.58–14.79). Steatosis showed no significant association with advanced fibrosis in any of these models (Table 4).

### 3.5. Frequency of Hepatic Steatosis

CAP-defined steatosis was present in 30 patients (24.0%). Mean CAP was 287.1 dB/m (SD 23.9) in patients with steatosis and 214.7 dB/m (SD 15.3) in those without (Figure 2A). Patients with and without steatosis did not differ in age, sex, HDV-RNA status, ALT elevation, oral NA use or previous peg-IFN-α therapy (Table 1). LSM was also similar (median 9.7 vs. 10.4 kPa, *p* = 0.188; Figure 2B), as were the proportions with LSM ≥ 9.5 kPa (66.7% vs. 71.6%, *p* = 0.649) and with cirrhosis (20.0% vs. 13.7%, *p* = 0.394). Among the 85 patients with detectable HDV-RNA, 20 (23.5%) had steatosis, and no significant association with advanced fibrosis was found (aOR 0.94, 95% CI 0.26–3.38, *p* = 0.918), although this estimate was imprecise.

## 4. Discussion

Few studies from Turkey have used FibroScan to look at fibrosis and steatosis together in a sizeable group of patients with HDV infection. Three findings stand out. About a quarter of patients had steatosis; no significant association between steatosis and LSM-defined advanced fibrosis was found; and detectable HDV-RNA was associated with advanced fibrosis. In addition, fewer than one in three patients treated with peg-IFN-α had achieved SVR.

### 4.1. SVR, Cirrhosis and HCC Burden

Of the 85 patients treated with peg-IFN-α, 30.6% had achieved SVR. This is somewhat higher than the pooled SVR of 19% reported for pegylated interferon in a systematic review [22], although comparisons are limited by differences in patient selection, treatment duration and the timing of HDV-RNA testing. Patients with SVR had lower liver stiffness and almost always normal ALT. This pattern is in line with long-term HIDIT-II data, in which HDV-RNA suppression was associated with fewer cases of HCC and decompensation [23], but our observation is descriptive: without pre-treatment LSM and ALT values, and with an unknown interval between treatment and FibroScan, we cannot tell whether these differences reflect treatment response or milder disease at baseline. Comparisons of virological response between studies are further complicated by limited standardisation of HDV-RNA assays, which show systematic differences in quantification [24].

The cirrhosis prevalence of 15.2% in our cohort is lower than in several other HDV series. Couto et al. found compensated advanced chronic liver disease, defined by LSM ≥ 15 kPa, in 57% of 77 patients from the western Amazon basin [16], despite using a higher threshold than ours. Differences in HDV genotype may contribute: genotype 1 predominates in Turkey, whereas genotype 3, which circulates in the Amazon region, has been associated with particularly severe disease [5]. Cohort characteristics, disease duration, treatment exposure and diagnostic criteria also differ, and our patients were relatively young. In a longitudinal German cohort, severe clinical events including HCC occurred in 11 of 49 patients [25], and a Turkish study found that peg-IFN therapy did not eliminate the risk of cirrhosis and HCC [26]. In our cohort, eight patients had a history of decompensation and two had HCC, which again points to the need for more effective treatment.

### 4.2. Limitations of Pegylated Interferon and Emerging Therapies

In the phase 2 LIMT-1 trial, peginterferon lambda produced a durable virological response in 36% of patients receiving the 180 µg dose [27], suggesting that interferon type and dose may influence outcomes. A more important recent advance has been bulevirtide (BLV), an entry inhibitor targeting the NTCP receptor, which received full European Medicines Agency approval in July 2023. In the phase 3 MYR301 trial, BLV was superior to delayed treatment at week 48 [17], and responses, including improvements in liver stiffness, continued through week 96 [28]. Real-world data from 108 Italian patients in the ARISTOTLE study were consistent with these results [29]. Bulevirtide was not available to the patients in our cohort.

Effective viral suppression may also change the metabolic picture. After cure of hepatitis C, steatosis is found in a substantial proportion of patients despite viral clearance, and changes in lipid metabolism are common [30]. Whether a similar shift occurs after HDV suppression with bulevirtide is unknown. In our cohort CAP was similar in responders and non-responders (median 231 vs. 217 dB/m, *p* = 0.322), although the interval since treatment was unknown and the design was cross-sectional. If steatosis persists once the viral component is controlled, it could contribute to residual fibrosis or HCC risk, which makes metabolic assessment a reasonable part of follow-up in treated patients.

### 4.3. Steatosis in Chronic HDV Infection

The 24.0% frequency of steatosis in our cohort cannot be compared directly with the roughly 30% prevalence reported in the general population [11], since the two populations are not matched for age, sex, body mass index or region, and we lacked data on metabolic risk factors. It is worth noting, though, that chronic HBV infection has been linked to lower rather than higher rates of steatosis [9,10]. Our data cannot show whether HDV modifies this relationship, but they do show that steatosis is common enough in HDV-infected patients to be worth recording. Cirrhotic and non-cirrhotic patients had similar CAP values (*p* = 0.915). This may mean that steatosis here is not simply a feature of advanced disease, but it may equally reflect reduced accuracy of CAP in cirrhotic livers [21], and the observation should be regarded as exploratory (Figure 2). Because cardiometabolic risk factors were not available, the steatosis we observed cannot be attributed to metabolic dysfunction. HDV infection itself, coexisting cirrhosis or other unmeasured factors may contribute to hepatic fat accumulation, so our data do not show that viral and metabolic liver injury coexist in these patients.

The negative findings also need careful interpretation. Even for advanced fibrosis, with 88 events, the confidence interval for steatosis (aOR 0.82, 95% CI 0.32–2.12) was wide, and it was wider still among patients with detectable HDV-RNA (aOR 0.94, 95% CI 0.26–3.38); a clinically meaningful association in either direction therefore cannot be excluded. For cirrhosis, however, there were only 19 events, and the confidence intervals for both steatosis (aOR 1.71, 95% CI 0.57–5.16) and detectable HDV-RNA (aOR 2.99, 95% CI 0.81–11.07) were wide. The absence of a significant association with cirrhosis therefore cannot be distinguished from a lack of statistical power and should not be taken as evidence that steatosis plays no role in disease progression. In addition, all fibrosis outcomes were defined by LSM cut-offs that have not been validated in HDV infection, and the regression results should be read with this in mind.

### 4.4. Strengths and Limitations

Among the strengths are a fairly large cohort for an uncommon infection, recruited from a high-prevalence region, and uniform LSM and CAP assessment in all patients. It also has important limitations. The design was cross-sectional and single-centre, so causal inferences cannot be drawn. We had no systematic data on body mass index, waist circumference or other cardiometabolic risk factors; steatosis was therefore defined by CAP alone, the 2023 Delphi criteria for MASLD could not be applied, and the metabolic origin of steatosis could not be established. Routine biochemical parameters other than ALT, HBV DNA, quantitative HDV-RNA and HDV genotype were not available for analysis; in particular, the absence of AST, platelet count and albumin prevented calculation of serological fibrosis scores such as FIB-4 and APRI, which could have complemented the FibroScan findings. LSM and CAP can be influenced by hepatic inflammation, and patients with elevated ALT were not excluded, which may have led to overestimation of fibrosis in some cases. No liver biopsies were performed, so steatohepatitis could not be diagnosed. With only 19 cirrhosis events, the cirrhosis model is exploratory and imprecise; no formal sample size calculation was made; and subgroup comparisons were not corrected for multiple testing. Finally, the interval between the end of peg-IFN-α therapy and FibroScan was unknown and varied between patients. Prospective studies with complete metabolic and virological data are needed, particularly in patients treated with bulevirtide.

## 5. Conclusions

About one in four patients with chronic HBV infection and persistent anti-HDV seropositivity in this cohort had CAP-defined hepatic steatosis. Detectable HDV-RNA was associated with LSM-defined advanced fibrosis, whereas no significant association with steatosis was found; the precision of this estimate was limited, and the exploratory cirrhosis analysis was underpowered to exclude a modest effect. Without cardiometabolic data, the metabolic origin of the steatosis could not be established, and non-invasive tools alone cannot separate metabolic from HDV-related liver injury. Whether steatosis influences long-term outcomes in HDV infection, particularly after viral suppression with new agents such as bulevirtide, warrants prospective evaluation with complete metabolic phenotyping.

## Figures and Tables

**Table 1:** Characteristics of patients with chronic HDV infection according to hepatic steatosis status.

Variable	All (*n* = 125)	Steatosis (−) (*n* = 95)	Steatosis (+) (*n* = 30)	*p*
Age, years, median (IQR)	39.0 (29.0–47.0)	38.0 (29.0–48.5)	39.0 (28.5–45.5)	0.592
Duration of HBsAg positivity, years, median (IQR)	9.0 (8.0–12.0)	9.0 (8.0–12.0)	9.5 (8.0–12.0)	0.572
LSM, kPa, median (IQR)	10.1 (9.1–10.7)	10.4 (9.3–10.7)	9.7 (9.1–10.7)	0.188
CAP, dB/m, median (IQR)	218.0 (211.0–239.0)	211.0 (211.0–226.0)	283.0 (269.0–306.5)	—
Male sex, *n* (%)	93 (74.4%)	72 (75.8%)	21 (70.0%)	0.632
Detectable HDV-RNA, *n* (%)	85 (68.0%)	65 (68.4%)	20 (66.7%)	1.000
ALT > ULN (F > 25, M > 35 U/L), *n* (%)	55 (44.0%)	39 (41.1%)	16 (53.3%)	0.293
On oral NA therapy, *n* (%)	20 (16.0%)	16 (16.8%)	4 (13.3%)	0.780
Prior peg-IFN therapy, *n* (%)	85 (68.0%)	63 (66.3%)	22 (73.3%)	0.511
Cirrhosis, *n* (%)	19 (15.2%)	13 (13.7%)	6 (20.0%)	0.394
LSM ≥ 9.5 kPa, *n* (%)	88 (70.4%)	68 (71.6%)	20 (66.7%)	0.649

Values are median (IQR) or *n* (%); *p* values come from the Mann–Whitney U test and Fisher’s exact test, respectively. Steatosis: CAP ≥ 248 dB/m; ALT > ULN: >25 U/L in women, >35 U/L in men; cirrhosis: LSM ≥ 12.5 kPa; NA: nucleos(t)ide analogue; peg-IFN: pegylated interferon. CAP was not compared statistically because it defines the steatosis groups.

**Table 2:** Characteristics of patients previously treated with pegylated interferon-alpha according to virological response (*n* = 85).

Variable	No SVR (*n* = 59)	SVR (*n* = 26)	*p*
Age, years, median (IQR)	36.0 (28.5–46.0)	38.5 (30.0–46.8)	0.785
HBsAg duration, years, median (IQR)	9.0 (8.0–15.0)	8.0 (8.0–11.0)	0.052
LSM, kPa, median (IQR)	10.4 (9.6–10.7)	8.6 (7.7–9.9)	<0.001
CAP, dB/m, median (IQR)	217.0 (211.0–241.0)	231.0 (211.0–269.8)	0.322
Male sex, *n* (%)	43 (72.9%)	19 (73.1%)	1.000
ALT > ULN, *n* (%)	35 (59.3%)	1 (3.8%)	<0.001
On oral NA therapy, *n* (%)	10 (16.9%)	3 (11.5%)	0.746
Steatosis, *n* (%)	14 (23.7%)	8 (30.8%)	0.593
LSM ≥ 9.5 kPa, *n* (%)	48 (81.4%)	11 (42.3%)	<0.001
Cirrhosis (LSM ≥ 12.5 kPa), *n* (%)	8 (13.6%)	2 (7.7%)	0.717

SVR: undetectable HDV-RNA at a post-treatment assessment at least six months after the end of therapy (study-specific definition). Statistics as in Table 1. Percentages are column percentages, i.e., the proportion of patients with each characteristic within the SVR and no-SVR groups.

**Table 3:** Multivariable logistic regression analysis of factors associated with advanced fibrosis and cirrhosis.

Variable	Advanced Fibrosis aOR (95% CI)	*p*	Cirrhosis aOR (95% CI)	*p*
Steatosis (CAP ≥ 248 dB/m)	0.82 (0.32–2.12)	0.684	1.71 (0.57–5.16)	0.338
Age (per year)	1.02 (0.98–1.05)	0.350	1.03 (0.99–1.07)	0.181
Male sex	1.14 (0.45–2.91)	0.789	1.39 (0.42–4.63)	0.593
Detectable HDV-RNA	4.89 (2.13–11.26)	<0.001	2.99 (0.81–11.07)	0.100

Advanced fibrosis: LSM ≥ 9.5 kPa (88 events); cirrhosis: LSM ≥ 12.5 kPa (19 events). aOR: adjusted odds ratio; CI: Wald confidence interval. The cirrhosis model is exploratory, and its estimates are imprecise because of the small number of events.

**Table 4:** Sensitivity analyses for LSM-defined advanced fibrosis (LSM ≥ 9.5 kPa).

Model	*n* (Events)	Steatosis, aOR (95% CI)	*p*	Detectable HDV-RNA, aOR (95% CI)	*p*
Main model (Table 3)	125 (88)	0.82 (0.32–2.12)	0.684	4.89 (2.13–11.26)	<0.001
+ALT elevation	125 (88)	0.79 (0.30–2.08)	0.638	4.12 (1.45–11.72)	0.008
+ALT elevation, previous peg-IFN-α, current NA therapy	125 (88)	0.81 (0.31–2.14)	0.673	4.22 (1.47–12.07)	0.007
Patients with normal ALT only	70 (43)	1.07 (0.29–3.89)	0.922	4.83 (1.58–14.79)	0.006

All models are adjusted for age and sex in addition to the variables shown. ALT elevation: >25 U/L in women, >35 U/L in men. aOR: adjusted odds ratio; CI: Wald confidence interval; NA: nucleos(t)ide analogue; peg-IFN-α: pegylated interferon-alpha.

## Data Availability

The data presented in this study are available on reasonable request from the corresponding author.
